# Composite Polymeric Sucker Rod Guides: State-of-Practice, Causes of Failure, and Circular Economy Opportunities

**DOI:** 10.3390/polym17212932

**Published:** 2025-10-31

**Authors:** Chundu Gyem Tamang, Allan Manalo, Paulomi (Polly) Burey, Wahid Ferdous, Tristan Shelley, Mayur Patel, Tony Chapman

**Affiliations:** 1 School of Engineering, Centre for Future Materials (CFM), University of Southern Queensland, Toowoomba, QLD 4350, Australia; allan.manalo@unisq.edu.au (A.M.); wahid.ferdous@unisq.edu.au (W.F.); 2 School of Agriculture and Environmental Science, Centre for Future Materials (CFM), University of Southern Queensland, Toowoomba, QLD 4350, Australia; polly.burey@unisq.edu.au; 3 Centre for Future Materials (CFM), University of Southern Queensland, Toowoomba, QLD 4350, Australia; tristan.shelley@unisq.edu.au; 4 Oilfield Piping System, Toowoomba, QLD 4350, Australia; mayur@oilpipesys.com (M.P.); tony@oilpipesys.com (T.C.)

**Keywords:** polymeric rod guides, glass fibre, thermal degradation, waste, polymeric wear resistance, contaminants

## Abstract

The oil and gas industry generates substantial amounts of polymeric waste each year, including sucker rod guides manufactured from premium thermoplastics such as Polyphenylene Sulphide (PPS), Polyacrylamide (PAA), Polyamide (PA), and Polyether ether ketone (PEEK). It is estimated that, annually, approximately 18,600 metric tonnes of polymeric sucker rod guides are discarded worldwide, contributing significantly to landfill accumulation. This paper critically reviews the behaviour of polymeric rod guides when exposed to downhole environments where high temperature, pressure, contamination, and severe mechanical stresses act simultaneously. These components are essential in maintaining system reliability, yet research and development on polymeric rod guides remain limited, and investigations into their degradation and failure mechanisms are non-existent. In addition, there are currently no established approaches for recycling or reusing worn polymeric guides, which restricts progress toward sustainability and contributes to the increased accumulation of polymer waste in landfills. This review highlights these gaps and discusses future research directions that could improve the performance and service life of glass-fibre-reinforced polymeric components, while also creating opportunities for recycling and circular economy.

## 1. Introduction

Polymers are widely used in the oil and gas industry, which represents the largest petroleum exploration and production sector [1]. This industry requires artificial lift systems to lift produced fluids to the surface [2,3]. While the environment, fluid properties, economics, availability of equipment, and reservoir depths are critical considerations in selecting the most suitable lift method, sucker rod pumps have been the most popular choice among operators [2,4]. In these, considerable occurrences of failures including tubing and sucker rod damage [5,6,7], which contribute to major production interruptions and costly replacements [8,9], are common problems.

Previous research studies [8,10] show that more than 50% of such failures are caused by the lack of or inefficiency of polymeric rod guides. Polymeric rod guides are mostly fibre-reinforced thermoplastics and have various downhole applications vital for increasing the system’s service life [11,12,13,14]. The use of such polymeric rod guides has often been linked to exceeding downhole production life and resulting in nullification of rod-related downtime [15,16]. Moreover, available research studies [17,18,19] show that polymeric rod guides have an important role in deviated wells with high dog-leg severity and side loads. These downhole applications mandate different configurations and design requirements.

Polymeric rod guides are designed according to field environments and well-bore geometry [8,11]. They are generally installed on sucker rods with varying configurations [11] and it is critical for them to be placed with the most suitable designs. Different polymeric rod guide designs include concave and convex types [8], in addition to a variety of styles such as straight, extended, slanted, and wide vanes. Polymeric rod guides can also either be directly moulded on sucker rods or be field installed [16]. Even though investigations on thermoplastic guide failures have determined Erodible Wear Volume (EWV) as the primary cause of making them unsuitable for service [8,10], there are no experimental justifications provided through characterisation tests. Furthermore, various types of contaminants such as Carbon dioxide (CO_2_), Hydrogen sulphide (H_2_S), Sulphur dioxide (SO_2_), Chloride (Cl), and Oxygen (O_2_) are known be present in the downhole environment [12,20]. Additionally, solid contaminants such as abrasive sand particles increase wear rates [17] and are important in studying polymeric rod guide failures.

Historically, sucker rod guides were fabricated from metallic materials such as steel or cast iron, which offered strength but caused severe friction and wear against tubing surfaces [16,21,22]. To mitigate these problems, the industry progressively adopted polymeric materials in the late twentieth century, beginning with unreinforced polymers and later incorporating thermoplastics such as Polyamide (PA) and Polyphenylene Sulphide (PPS) [23]. These developments significantly improved wear resistance and corrosion protection [16,24]. Over time, the transition to glass-fibre-reinforced thermoplastics marked a major advancement, offering higher strength-to-weight ratios and extended service life [8,25,26]. More recently, high-performance polymers such as Polyether ether ketone (PEEK) and Polyphthalamide (PPA) have been introduced to meet the increasing thermal and chemical demands of deeper wells [27,28]. This chronological evolution underpins the current state-of-practice in the use of composite polymeric rod guides in the oil and gas industry.

High performance thermoplastics have been widely used for downhole components due to their high strength, corrosion resistance, and thermal stability [25]. Prior studies have reported that PPS and PEEK offer exceptional wear and chemical resistance under sour and CO_2_ environments, whereas polyamides are more moisture sensitive but cost-effective [27,29]. These material specific behaviours are critical in understanding polymeric rod guide performance and form the basis for the comparative review presented.

With oil resources making up one-third of the global energy demand [30], this adds concern to the existing problem of plastic production which is projected to reach 30,000 million tons by 2050 [31,32] while <10% is being recycled [33,34]. The plastic industry, which generated 4.5% global greenhouse gas emission in 2015 [35,36], is projected to emit 6.5 Gt CO_2_ eq by 2050 [37]. Moreover, the global plastic production was 400.4 million tonnes in 2022 [38] and according to the Statistical Research Department, it is projected to reach 445.25 million tonnes in 2025. This massive plastic waste remains a major environmental concern [39,40,41]. However, the oil and gas industry is still unable to find sustainable pathways for used polymeric rod guides. Moreover, for a polymer’s rod guide application, the relationship between type of thermoplastic, effect of glass fibre reinforcement, and mode of failure is still not understood. Hence, like the existing waste management schemes which mostly practice landfilling and incineration [42], oil and gas industries have also simply opted to continuously landfill polymeric rod guides. Therefore, there is a need for a better understanding of these polymeric guides’ current applications and causes of failure.

Accordingly, this review is guided by the following key research questions:

(1) What are the current performance limitations of polymeric sucker rod guides used in oil and gas wells?
(2) What are the dominant degradation and failure mechanisms affecting their service life under downhole conditions?
(3) What potential recycling and circular economy pathways can be developed for these polymeric components to support sustainability within the oil and gas industry?

Addressing these questions will provide a scientific framework that distinguishes this review from general summaries and support future research and development directions for polymeric sucker rod guides.

Therefore, this review provides the first comprehensive discussion that links the failure behaviour of polymeric sucker rod guides with the principles of sustainability and circular economy. Previous publications have usually treated the topic only from a mechanical or operational point of view. This review discusses the degradation of high-performance thermoplastics under downhole conditions and critically analyses possible reuse and recycling pathways. By combining these two perspectives, the paper outlines a new approach for extending component life and reducing polymeric waste in oilfield operations.

## 2. Sucker Rod Pumping System

**Figure 1:**
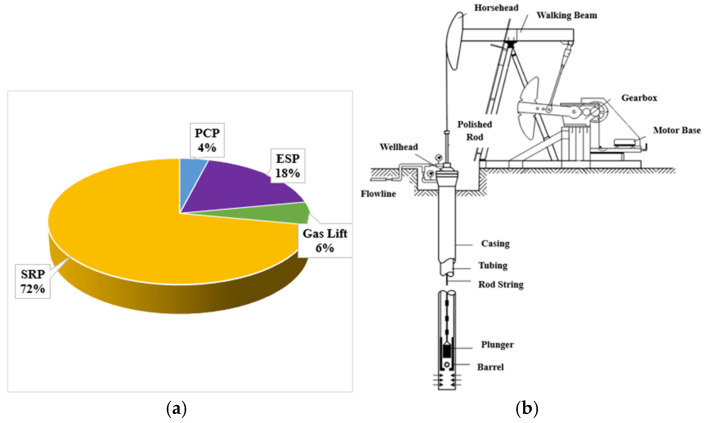
(**a**) Share of different artificial lift systems, illustrating the relative dominance of sucker rod pumping worldwide; (**b**) components of a sucker rod pumping system showing surface and downhole elements. Source: (**a**) [45], licensed under Creative Commons Attribution License; (**b**) [44], licensed under Creative Commons Attribution License (http://creativecommons.org/licenses/by/4.0/).

The inception of a petroleum reservoir’s utility begins with high productivity where fluid lift occurs spontaneously by natural lift. However, due to the reservoir’s pressure decay, it becomes necessary to use artificial lift technologies [3]. The sucker rod pumping (SRP) system is one of the most widely used artificial lifting systems around the world [6,43]. It represents more than 70% of the total lift technologies as depicted in Figure 1a. This is mainly due to its easy application method, low capital, and operational costs [2,43]. A typical sucker rod pumping system is categorised into surface and sub-surface components. Components at the surface include a motor, gearbox, pumping unit, polished rod, and wellhead while the sub-surface consists of rod string, tubing, rod guides, and the downhole pump as depicted in Figure 1b [44].

### 2.1. Failure of Sucker Rod Pumping Systems

Romero and Almeida [3,23] described rod string, which is several thousand metres in length and comprises connected sucker rods, as a very important downhole component providing linkage between the surface and sub-surface units of the pumping system. If a single component of rod string fails, it could result either in leakage within the string or cause complete string failure [2]. The failure of rod string, which behaves like a slender bar, also results in total production loss and affects the key performance indicators (KPI) such as energy performance, financial performance, and total efficiency [46,47].

**Figure 2:**
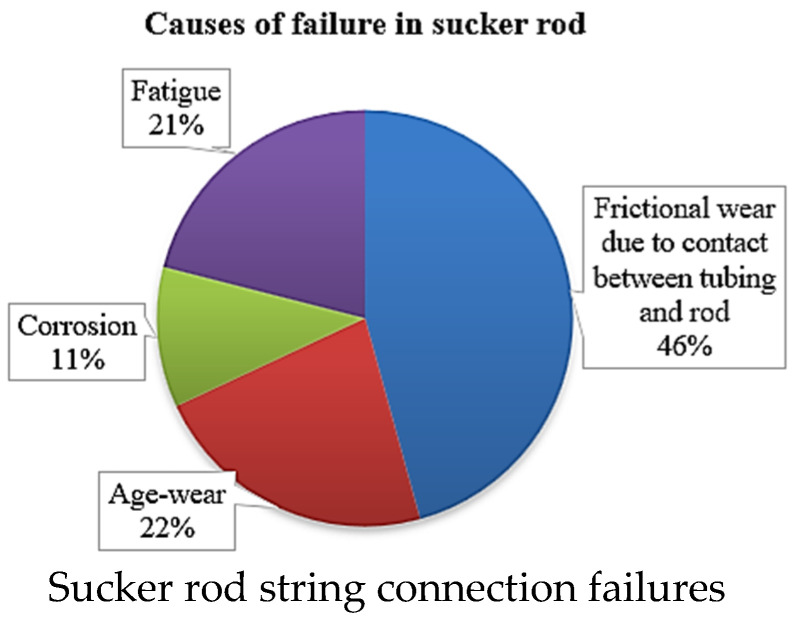
Primary causes of sucker rod string and connection failures, including age-wear, fatigue, and corrosion effects, illustrating how repeated load cycles lead to mechanical damage. Source: Author’s own design using data from [46].

Another cause of mechanical failure, which has been explored by several researchers, includes the collision between downhole rod string/coupling and tubing, which is the pipe through which produced fluid traverses [2,23]. This causes severe breakages, leakages, and mechanical damage due to erosion and wear [48,49] of the weakest parts [18] promoting rod string and connection failures [46] as shown in Figure 2.

**Figure 3:**
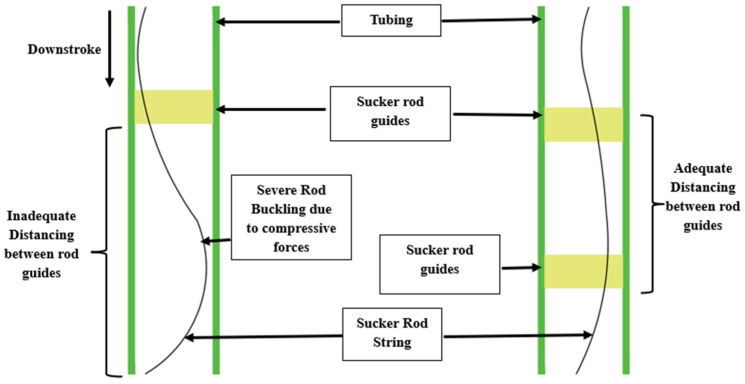
Effect of sucker rod guide spacing on downhole rod-tubing collision. Inadequate spacing causes compressive buckling and contact with tubing, while optimal spacing maintains alignment and reduces wear. Source: Author’s own design based on data from [11].

This collision primarily occurs in deviated or horizontal wells where the rod string deflects from the tubing axis due to gravitational sagging, side loads, or compression-induced buckling [11,12,14]. The contact between the coupling and tubing’s inner wall leads to localised abrasion and accelerated wear, especially under dynamic loading and corrosive downhole fluids [49]. To reduce such mechanical contact, centralising components such as polymeric rod guides are installed along the rod string to maintain concentric alignment and evenly distribute wear [18], as illustrated in Figure 3.

**Figure 4:**
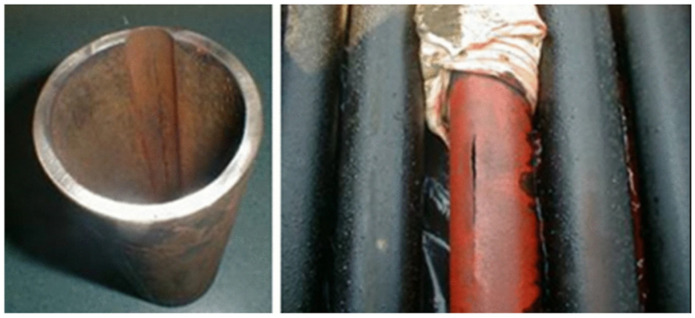
Typical tubing wear patterns caused by rod-tubing contact and abrasive motion, highlighting characteristic wear zones along the tubing wall. Source: [2], licensed under CC BY 4.0.

Figure 4 illustrates an eroded tubing pipe due to friction caused by the metal-to-metal contact between sucker rod coupling and tubing in case of inadequate distancing between rod guides. In addition to failures caused by frictional forces, the dynamic behaviour of the pumping system puts the rod string under compressive loads and causes buckling effects and further wear [50], especially in deviated wells [4]. Other operational failures include pressure differential problems, electrical failures, temperature failures, and gas problems which affects the overall efficiency of downhole equipment [4,5,7].

### 2.2. Use of Polymer Sucker Rod Guides to Mitigate Downhole Failures

**Figure 5:**
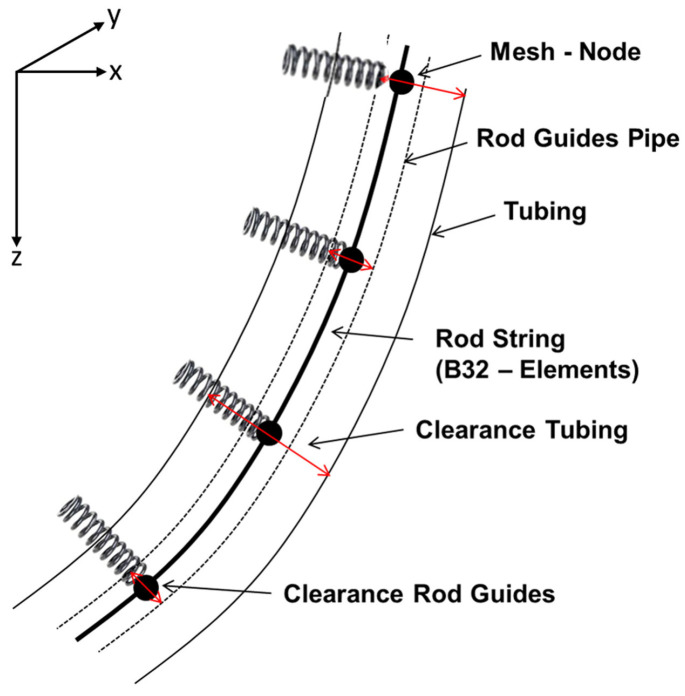
Definition of downhole contact between rod and tubing, showing the geometric relationship used to evaluate side loading and friction with the red lines indicating the radial clearance between the rod string or rod guides and the tubing. Source: [45], licensed under Creative Commons Attribution License.

Polymer rod guides are primarily used for sucker rod string centralisation [51], prevention of contact between rod string/coupling and tubing [11,12,14], rod buckling aversion [15], corrosion resistance [13], and paraffin control [16]. The use of glass-fibre-reinforced polymeric-moulded composite rod guides with high-performance additives, provides an effective solution to overcome rod failures and tubing leakage problems as suggested by Narso [24]. This prevents contact between the rod string and inner tubing wall [8,11], which lessens friction between downhole equipment and enhances its service life by centring the sucker rod string. For example, as shown in Figure 5, the use of polymer rod guides can reduce the impact of tubing and rod string buckling and provides optimisation of existing equipment for economic improvement [8].

**Figure 6:**
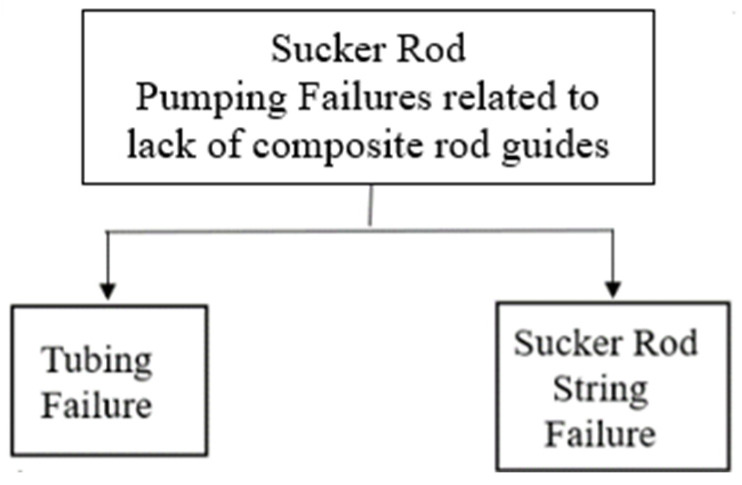
Typical failure modes in a sucker rod pumping system, including tubing and sucker rod string failures, summarising the main mechanical issues encountered in operation. Source: Author’s own design with information from [8].

For example, a research study evaluated a group of mature fields in South Sumatera in Indonesia, experiencing tubing leaks and parted rods due to the metal-to-metal friction [24]. This failure resulted in serious production downtime and caused 17,000 oil barrel losses and an expenditure of 200,000 USD. Consequently, they found that polymeric-moulded rod guides reinforced with high-performance additives extended and even exceeded the average run life of 3 wells in which they were installed. Prevention of metallic friction through polymer rod guide installation is crucial as indicated in Figure 6, since tubing leaks or tubing failures contribute to 59% of pull breakout reasons. This is according to a study evaluating 58 wells conducted by Dove and Smith [9] which found that more than 50% of the failures are related to lack of polymer rod guides [8].

Narso [24] reported that moulded-on polymer guides used in 3 different wells exceeded the downhole service life and exhibited zero rod-related downtime. In another study, immature tubing, sucker rod, and coupling failures occurred where no polymer guides were installed with a predicted side load (SL) of 0.18 kN force [11]. In contrast, there were no failures observed in locations where guided sucker rods were installed. Polymer guides also perform the function of breaking clogs (paraffin precipitation) from the tubing wall [8] which in turn improves the produced fluid’s flow rate. Similarly, according to Nurmohamed et al. [52], 54% of the average annual 580 downhole failures between 2008 and 2012 were found to be due to tubing leaks. These failures were caused by rod-tubing wear with up to 70% of tubing leakages occurring on the lower tubing string. An evaluation of 8 well pilot programs in wells with maximum tubing failures with the installation of polymer guides, found that the work-over frequency in 4 wells was reduced from 5 to 3 jobs annually (40% reduction). On the other hand, the remaining 4 wells undergoing production were without any tubing failures.

**Figure 7:**
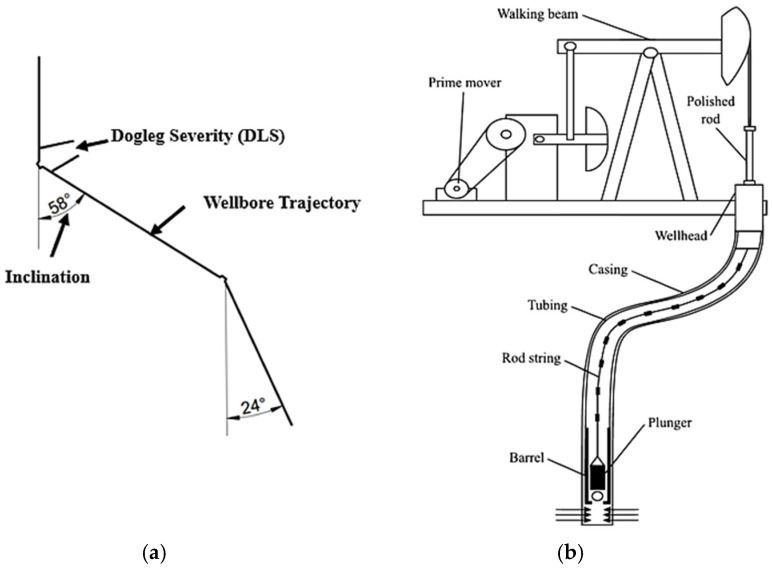
(**a**) Application of sucker rod guides in deviated wells, illustrating how guides maintain rod alignment; (**b**) schematic of a directional guided sucker rod well showing guide placement and trajectory control. Source: (**a**) Author’s own design; (**b**) [54], licensed under Creative Commons Attribution 4.0 International License.

Moreover, polymer guides also have a critical function in highly deviated wells [11]. The unpreventable bending of the rod string and production tubing causes disruptions to the rod string centralisation which makes it encounter tubing at different depths. Hence, polymer guides are used at depths with high loads and forces. These guides made of reinforced plastics are used as bearings between rod string and tubing [49]. For example, a study on dog-leg severity (DLS) [53], indicated that well deviations of 0 to 3 degrees/30.48 m are considered non-problematic while deviations of 3 to 5 degrees/30.48 m are known to cause increased wear and friction. Deviations of >5 degrees/30.48 m are not recommended since it is evidenced to cause problems such as increased replacement costs and system downtime. Nonetheless, research has shown that such wells with higher deviations can still be operated with the use of important tools such as centralisers or polymer guides [18] as shown in Figure 7. This assists in servicing the rapidly increasing number of deviated wells [19] where contact loads could become more significant [17].

For example, an evaluation of ConocoPhillips coal bed methane gas wells from San Juan Basin, which were shallow (<1219 m), indicated that no guides were needed according to conventional practices. However, DLS and SL resulted in hole in tubing (HIT) failure at 503 m in a constant build well section with SL of <22.7 kg. Rods were found to be in constant contact with tubing even though SL was low. Subsequently, polymer guides that were then installed in the constant build sections allowed SL of around 90.7 kg equalling 10 years of possible run time [53].

Similarly, a study on PCP systems [18] found that in a shallow onshore field (Bhagyam Field), 58% of the total failures were due to rod failures and 16% due to tubing failures. To mitigate these, non-rotating guides and snap-on guides were used in addition to other methods which decreased cyclic loads on the rod string and maximised rod run life by 27%. Goyal et al. [55] investigated stress loadings in ten deviated SRP wells and reported that the majority of failures involved worn sucker rods and cracked tubing, with three polymer guides found damaged in one of the studied wells.

**Figure 8:**
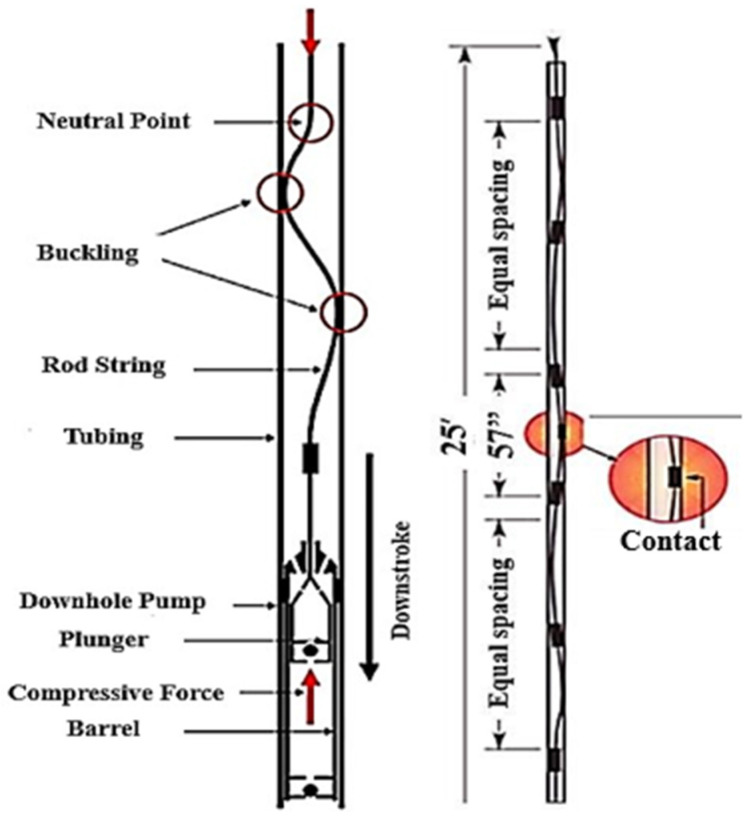
Sucker rod buckling during upstroke and downstroke compression and its impact on contact forces and wear distribution along the rod string. Source: [2], licensed under Creative Commons Attribution 4.0 International License.

This study recommended the application of moulded polymeric guides which can prevent or minimise rod string and tubing failures. According to Jiang et al. [56], the use of polymer guides/centralisers is non-negotiable in deviated wells, and helps reduce replacement and repair costs. Hence, these studies highlighted the importance of using polymer guides in oil and gas pumping systems. For example, the use of a composite polymeric guide during buckling is given in Figure 8 [2]. It is therefore important to understand the operation and use of polymer guides to mitigate downhole failures.

## 3. Characteristics of Polymer Rod Guides

Polymer rod guides are predominantly made of thermoplastic polymers reinforced with glass fibres and are attached to sucker rods at different depths, especially at locations of previous high tubing wear. There are several variations in the lengths, shapes, materials, and designs used in manufacturing polymer guides depending on the field environment and well-bore geometry [8]. According to a study by Clarke and Malone [11], through the use of rod guiding software, past failure data, and considering the well-bore geometry, side load, and diameter of sucker rod taper, different types of polymeric guide materials and configurations can be selected. These important parameters are discussed in detail in the following sub-sections.

### 3.1. Location of Polymeric Rod Guide Installation

**Figure 9:**
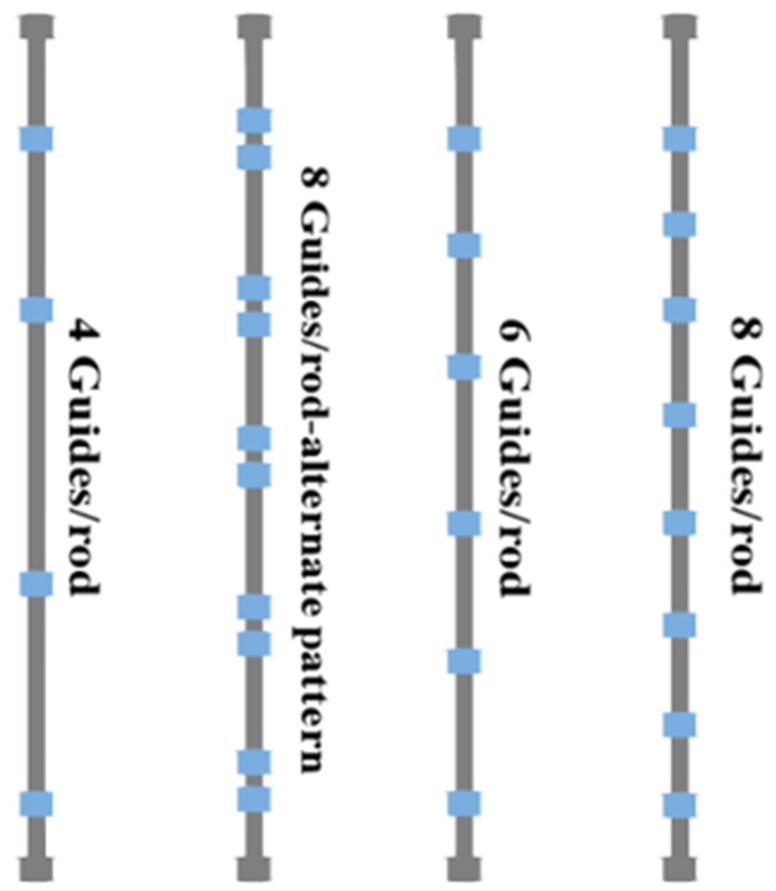
Different polymeric rod guide configurations used in sucker rod strings. Source: Author’s own design based on data from [11].

The service life of polymer rod guides depends on the location they are installed at. The guides are becoming exposed to more hostile environments with increasing well depths, where they need to have increased chemical, temperature, corrosion, and stress compatibility. On an average, six rod guides are used on each sucker rod of 7.62 m length. The configuration can, however, vary according to the design requirements, with some wells using as many as ten guides per rod while others only need as few as one guide per rod. According to a study by Hein and Rowlan [53], 22 kg SL per rod guide can be used for conventional designs and up to 10 guides per rod can be used for high SL. This would allow wells with 45.36 to 90.72 kg SL to be operated for more than 10 years just by using four guides per rod. Some of the common polymer guide configurations adopted by the industries are reflected in Figure 9 [11].

The specific positions of installation on sucker rods stabilises the radial motion of the rod string and prevents buckling and contact with tubing [8]. Therefore, polymer rod guides can extend the system’s run life if the correct spacing, number of guides per rod, and precise position on sucker rods are used. Moreover, calculation of the side forces exerted on or by the rod string can determine the guide’s location which is a critical parameter. This figure shows that various guide placement strategies are inclusive of design considerations for side loads and dog-leg severity. Additionally, the recommended number of polymer guides per rod for complete paraffin removal is calculated using the following formula:

$$\frac{Rod\;length\;}{Strokes\;}+1=Number\;of\;polymer\;guides\;per\;rod$$

### 3.2. Design of Polymer Rod Guides

**Figure 10:**
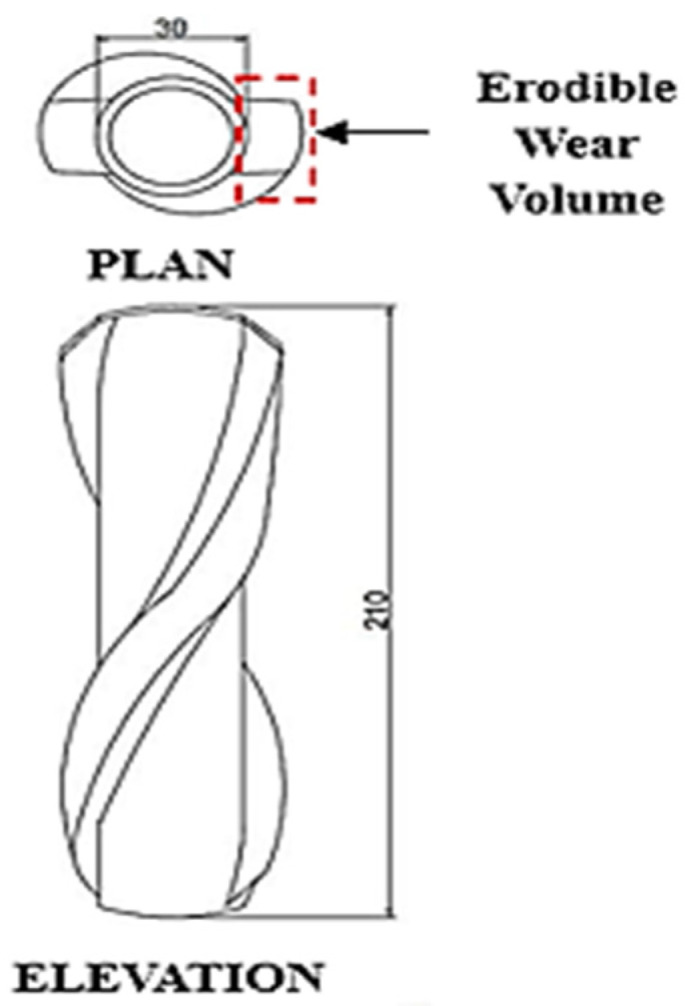
Plan and elevation of a polymer rod guide showing the erodible wear volume (EWV) and wear-interaction zones that influence guide longevity. *Source:* Author’s own design in AutoCAD 2019.

In general, there are two different types of polymer rod guides, mould-on and field installable. Mould-on polymer guides are pre-installed onto the sucker rods [8]. However, the functional efficiency of polymer rod guides depends on the volume of material that is at disposal to be eroded. This is known as Erodible Wear Volume (EWV) and it is larger than the outermost coupling diameter as shown in Figure 10 [8,11,16]. Polymer guides which act as centralisers in the downhole section have four primary types of categories of which ‘nylon, fixed, two-disc, and roller’ is an example. While the nylon, fixed, and two-disc types make use of sliding friction, the roller type uses rolling friction [56]. In some wells with lower side loads, roller guides are used. However, the wheel axles can break due to increasing loads, wear, and corrosion. Nonetheless, for higher side loads and temperatures stiffer materials can be selected [12]. Furthermore, depending on field requirements, polymer rod guides can either be concave or convex. For example, for a larger EWV a concave rod body with straight vanes can be used, and for a more significant fluid flow area convex guides can be used.

Another design criteria for effective guide functionality is its vane type which can either be straight or slanted. Straight vanes are used in low corrosion wells with one pull per year or less and can result in better performance with rod rotators and decrease fluid turbulence. Meanwhile, slant vanes which have wider cutting action effectively get rid of moderate paraffin and clean almost all of the tubing’s circumference [16]. According to Weatherford International, use of cobra rod guides with wider vanes allows greater EWV with enhanced protection.

Takacs [16] noted that only one side of a polymer guide contacts the tubing at a time, which may reduce its overall efficiency. To mitigate this, rod rotators are commonly used to promote uniform wear across the guide surface, thereby extending its operational lifespan. However, even though rotators are used, polymer guides can lose their efficiency in scraping paraffin from the tubing walls. This is often mitigated by adopting wider radial ribs which improve the scraping circumference. On the other hand, this increased cross-sectional area can reduce flow paths and decrease productivity. Similarly, for enhanced grip on the rod string, the polymer guide’s length is increased but it can cause drag or constriction on the produced fluid’s flow rate.

The different types of rod guides can also include wheeled guides, metal guides, snap-on guides, and moulded guides as shown in Table 1. Earlier, metal rod guides were popularly welded onto rods [16]. Spiral metal guides were also tested as a potential rod guide material, but it resulted in escalated friction, thereby increasing loads and stresses in the sucker rod string and causing rod failure due to metal-to-metal contact [21,22]. Wheeled guides set at 45 degrees to each other can also be used for centralisation and reduced frictional movement.

Snap-on guides, which are also known as field installable guides, are polymer based and used in centralising the rod string. A 30% to 40% increase in run life was predicted through simulations with the use of snap-on guides as evaluated by Khadav et al. [18]. However, this type of guide has a major limitation of not being secured on the rods, which allows it to float through the rod string’s length. This causes the guides to go missing and fall onto pump tops, thereby restricting rotor installation, which in turn causes larger cost implications. These types of rod guides are cheaper due to the absence of factory mounting costs and are generally used in wells with minor wear problems and lower downhole temperatures [16].

The most popular, reliable, and recent type of rod guide is the injection-moulded polymer guide reinforced with glass fibres, making it a composite thermoplastic guide material. These guides are manufactured using polymeric materials such as nylon [16,24] and have varying glass contents to enhance their mechanical properties.

Whilst the adoption of different rod guide types continues to be debated, the design of polymer rod guides needs to be analysed and selected with accuracy, precision, and field requirements so that it can perform its technical limits for an extended service life.

### 3.3. Literature Review on Polymer Rod Guide Materials and Types

A critical component of rod guide efficiency is the choice of parent material and combination of additives which largely affect the run time. This choice must be checked for compatibility with different well fluids [16]. As studied by Clarke and Malone [11], use of the right material, correct model, and precise configuration is important for maximum efficiency. These parameters along with manufacturing quality are vital in ensuring that the polymeric guides can fulfil their technical specifications.

Recent studies on glass-fibre-reinforced thermoplastics highlight significant differences in thermal stability, toughness, and wear behaviour between different thermoplastics. Polymer composites with fibre reinforcement are great alternatives to conventional metals due to their high strength-to-weight ratio and enhanced properties [25]. Hence, most of the sacrificial composite rod guides are made using polymers including Polyphenylene Sulphide (PPS), Polyacrylamide (PAA), Polyamide (PA), and Polyether ether ketone (PEEK) [8,26] with varying glass fibre reinforcements.

PA6 and PPS are widely used where a balance between cost, wear resistance, and ease of moulding is required, whereas PEEK is preferred for high-temperature or corrosive environments because of its superior chemical and thermal stability [16,28,57]. Glass-reinforced PA and PPS are used for higher temperatures and corrosive environments [16]. Glass fibre reinforcement typically enhances stiffness and abrasion resistance but can also reduce impact toughness and complicate recycling because fibre breakage shortens effective fibre length during melt processing [58,59,60].

According to Neustadt and Doppelreiter [20], rod guides fabricated using PA demonstrate excellent wear resistance, anti-frictional properties, and resistance to oil attacks while operating at temperatures above 100 °C. Furthermore, PA has a wide range of high strength applications in different industries due to its heat, wear, and chemical resistance as well as flexibility [57,61]. PPS is also referred to as an important thermoplastic with high strength and applicability in high-temperature technological applications [29].

**Figure 11:**
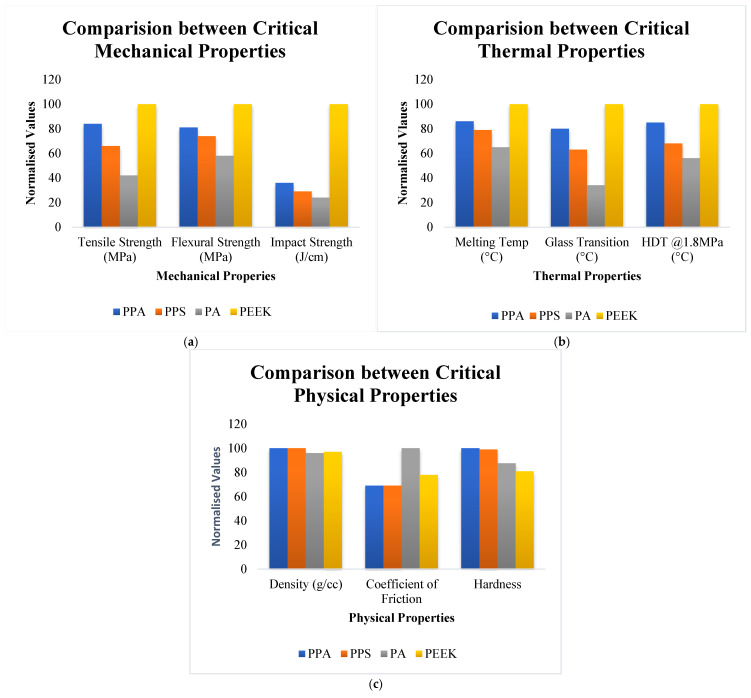
Comparative normalised properties of polymeric rod guide materials including PPA, PPS, PA, and PEEK. (**a**) Normalised mechanical properties (tensile strength, flexural strength, and impact strength); (**b**) Normalised thermal properties (melting temperature, glass transition temperature, and heat deflection temperature at 1.8 MPa); and (**c**) Normalised physical properties (density, moisture absorption, and hardness).

On the other hand, the qualities of PEEK, such as toughness, stiffness, chemical resistance, and high-temperature stability, add an advantage to its applicability [27,28] as one of the most high-performing in the PEK family [62,63]. Similarly, PA6 which is one of the most commercially popular polymers with amide linkages is often used in applications involving injection moulding [64]. Hence, reinforced thermoplastics are one of the most swiftly advancing class of composites [65] which have critical applications as rod guides in oil and gas exploration. Since there are different preferences on the most efficient materials, a comparative analysis of different composite rod guide materials with up to 35% glass fibre reinforcement is given in Table 2 and Figure 11a–c.

According to the comparative analysis in Table 2, PPS and PEEK have excellent resistance to hot oiling, including crude oil and are the best options in well environments involving oil exposure. PA6’s resistance to oil can often be affected at high-temperature settings. PA12 is known for its resistance to hydrocarbons which makes it a suitable choice for sweet crude exposure. It is also important to note PEEK and PPS’s exceptional resistance to chemical attacks from sulphur compounds in sour crude. However, PPA, PA12, and PA6’s resistance can be varied depending on sulphur content and other operating conditions. With respect to sand particles, PEEK and PPS can withstand abrasive wear due to good resistive properties but PPA, PA6, and PA12’s resistance can be affected by prolonged exposure and cause material degradation over time. Rod guide materials such as PA12, PPS, PPA, and PEEK do not easily absorb moisture and have good dimensional stability but PA6’s hygroscopic nature can make it swell and lose strength and have significant impacts on its morphology and structure [70]. PPA and PA6 also need to be evaluated for compatibility with prolonged brine solution exposure [16,26].

### 3.4. Regulatory and Performance Standards for Polymeric Rod Guides

Although there is currently no universal regulatory standard specifically governing the mechanical performance of polymeric sucker rod guides, several standards outline relevant benchmarks for materials used in downhole components. For example, the American Petroleum Institute (API) RP 11AR (2000) [71] provides general recommendations for sucker rod string design, including wear resistance and dimensional tolerance criteria. Additionally, the mechanical and thermal performance of polymeric rod guides is based on ASTM and ISO standards applicable to polymeric components. This includes ASTM D638 [72] and ISO 527 [73] for tensile properties, ASTM D790 [74] and ISO 178 [75] for flexural strength properties, ASTM D256 [76] and ISO 180 [77] for impact resistance, and ASTM D-648 [78] and ISO 75 [79] for heat deflection temperature.

Commercial products currently used in the Australian oil and gas industry demonstrate performance levels consistent with these requirements. Oilfield Piping Systems (OPS) which is an Australasian manufacturer and distributor of downhole materials based in Brisbane and Toowoomba, QLD, Australia, uses Duralon N6 25G HS BK106L, a heat-stabilised, 25 wt.% glass-fibre-reinforced Nylon 6 compound designed for use as polymeric rod guides. According to the company’s product data sheet (Duromer 2014) [80], the material exhibits a tensile strength of 120 MPa (ISO 527-2/1A) [73], flexural strength of 190 MPa (ISO 178) [75], impact strength of 6 kJ/m^2^ (ISO 180/1A) [77], and a heat deflection temperature of 204 °C (ASTM D648) [78].

Therefore, such industry examples emphasise that standardised testing and material qualification are important in ensuring the mechanical reliability and longevity of polymeric sucker rod guides under demanding oilfield conditions.

### 3.5. Manufacturing Techniques for Polymeric Rod Guides

Polymeric sucker rod guides are typically produced using conventional thermoplastic processing methods that enable dimensional control and consistent mechanical performance [81]. Injection moulding is the most common technique, allowing complex geometries and high-volume production of fibre-reinforced composites such as PPS, PA, and PEEK [82,83]. In this process, polymer granules compounded with glass fibres are melted and injected into precision moulds, ensuring uniform fibre dispersion and high interfacial bonding strength. For applications requiring stronger adhesion to the rod body, overmoulding [84] or insert moulding techniques are often used [85].

Compression moulding and extrusion are also used for specific designs and strength requirements [86,87]. The choice of processing technique directly affects fibre orientation, void content, and surface finish, all of which significantly influence wear behaviour, dimensional stability, and service life under downhole conditions [8].

Another important technique for fabricating polymeric rod guides is hot melt extrusion, which can be used to produce continuous profiles or semi-finished sections that are later machined into the required vane geometries [88]. Hot melt extrusion allows better control of fibre orientation, polymer melt homogeneity, and dimensional consistency, which are essential for achieving uniform mechanical properties and wear resistance [88,89]. This technique is particularly effective for thermoplastics such as PA, PPS, and PEEK, and can serve as a cost effective alternative to injection moulding for small batch or customised production [90,91,92].

## 4. Polymer Rod Guide Failure

The available literature [8,10,14] states weight and diameter loss as the main cause of guide failure. Polymeric rod guides suffer from this type of failure after being subjected to thousands of hours of operation and travelling thousands of kilometres in distance during the pumping process. As described by Clarke and Malone [11], EWV, which is an important indicator of rod guide performance [16], is the amount of polymeric material which is available to be eroded until the metal-to-metal contact between the sucker rod coupling and tubing wall takes place. It is the volume of guide that is required in between the largest outer diameter of rod string (coupling) and inner diameter of tubing.

Polymer rod guides can become embrittled, cracked, and in some cases end up with chunks breaking off whilst travelling within the well. If failures occur prematurely, they can cause workovers and well downtime, leading to production and cost losses for the site operator [11]. Another possible failure as described by Davis et al. [12] is interfacial wear due to the sliding of the polymeric rod guide against a hard counter face. This operation causes deformation of the polymer due to the adhesive forces and increased temperatures near or above the Vicat softening point. Increasing heat hazards with deeper mining depths has long been a cause for concern [93] and while this failure is related to temperature, studies on polymer rod guides do not provide further details on the effect of thermal degradation on its properties.

In a study involving sucker rod pump repair data [9], an evaluation of pumping component replacement showed that “worn” polymer guides fail the most often with the highest replacement frequency (more than 80%) among other sucker rod pumping system’s equipment failures.

Polymer rod guides often suffer one-sided abrasions because only a maximum of two guide fins are in contact with the inner tubing wall at any given time [8]. An effective solution to mitigate this problem is with the use of rod rotators, enabling even wear distribution and subsequently improving the service life of polymeric guides. Clarke and Malone [11] highlighted, however, that even though rod rotators are used, discrepancies still continue to be observed. For example, there is even wear distribution on guides located at the upper well section, uneven and one-sided wear at the middle section, and severe physical disfigurement and mechanical damage on rod guides at the bottom well section. This is because of the frictional forces between the sucker rods, tubing, and casing resulting in the ineffectiveness of rod rotators. The “stick slip” operation, which is a result of built-up torque energy being released, causes the polymer guides to aggressively encounter tubing, leading to significant damages.

### Wear Characteristics of Polymer Rod Guides

As investigated by Clemens et al. [8], wear behaviour of polymer composite rod guides is affected by sliding velocity, temperature, surface geometry, contact pressure, and type of produced fluid. It is also affected by both friction and tubing wear and can be divided into four major categories, namely, abrasive, adhesive, fatigue, and corrosive wear.

One research study was undertaken [10] which focused on an experimental investigation on the failure analysis of polymer guides. Their work consisted of a self-made testing facility incorporating two different conditions. Concave rod guides were used on a rod string which was approximately 240 mm long and 60 mm wide, that was centralised. The set-up also included a slightly worn tubing. The results of the experiment showed that for condition-1 of 20 °C, 6340 g weight on the test string, and 58 h of effective duration, the polymeric rod guide travelled a total distance of 104.34 km and lost 3 g of polymeric weight. On the other hand, for condition-2 of 60 °C, 5048 g weight on the test string, and 126 h of effective duration, the guide lost 2 g of polymeric weight due to erosion after travelling 227.04 km. This elaborate experiment suggests that the effect of normal force is more significant than the effect of temperature on a polymeric guide’s service life even though the operating temperature exceeds the glass transition temperature of the thermoplastic. The size reduction in the guides took place through the loss of polymeric material in millimetres until the guides failed to perform their designed functions.

In this experiment and a study by Clemens et al. [8,10], EWV is considered vital in assessing polymeric guide failure due to wear. However, the polymer used in rod guides have only been disclosed as nylon which inhibits the relevancy of wear rate results. Additionally, the experiment does not consider the existence of various types of downhole contamination which can affect wear rate. The study by Permanschlager [10] addresses the issue of rod guide disposals after a predefined time interval without assessing its actual condition. This can contribute to massive waste problems since each of these thermoplastics have a huge production rate. For example, PA had a global production of 6.2 million metric tons in 2022 [94]. However, this research only presented the outcome coefficients for friction and wear rate of polymeric rod guides under downhole conditions. There is a lack of characterisation of used polymeric rod guides to evaluate the microstructural, chemical, and thermal properties that will help explain how the aggressive downhole environment is affecting their degradation mechanisms and changing polymer properties, such as modulus, strength, and ductility [95].

Clarke and Malone [11] reviewed various polymer guide materials and suggested their failure could be a result of the used material’s fluid compatibility and temperature rating. This is because the polymer guides could potentially swell in a high-temperature and high-gravity oil environment, with the bottomhole temperature of some wells reaching the material’s technical limit of heat deflection temperature. This study documented that the only current practice being adopted is visual inspections for rod guide replacements which take place during workovers. They further recommended considering common oilfield chemicals and corrosion inhibitors while choosing rod guide materials. It was also noted that materials which can function in shallow wells at lower downhole temperatures can become ineffective when used at higher bottomhole temperatures and in deeper wells [53]. Additionally, increase in mining depth also results in higher crustal stress and gas pressure with the possibility of gas outburst [96,97]. Moreover, the temperature of the reservoir, brine composition, and exposure to oil are all factors which can affect a polymer’s permeability and subsequent retention [98]. Nonetheless, no research has considered these critical parameters when investigating polymeric rod guide failure.

## 5. Effect of Contaminants on Thermoplastics

Solid contaminants are mostly fracturing sand from reservoir stimulation which causes a significant increase in the wear rate of polymer rod guides [17]. A few wells are also subjected to sanding up when solids are released by the reservoir into the well-bore, which in some instances requires a clean out. Scale is also known to deposit on sucker rods, tubing, and other pumping components [11,55].

Research studies on the mitigation of downhole corrosion and wear failures [12,20] listed Carbon dioxide (CO_2_), Hydrogen Sulphide (H_2_S), Sulphur dioxide (SO_2_), Chloride (Cl), Oxygen (O_2_), microbiological, and galvanic corrosion as some of the most common downhole corrosion failures. Takacs [16] indicated that the contamination affecting the service life of downhole components is due to the downhole environment consisting of salt water in addition to corrosive gases. This causes corrosion in the form of pitting, resulting in local material loss at the surface, galvanic corrosion, hydrogen embrittlement, and sulphide stress cracking. Abrasive, adhesive, erosive, fretting, corrosive, and interfacial wear on polymeric guide materials can also be due to these contaminants. These are often identified as common wear problems and can result in either gradual or sudden failures. Additionally, increasing mining depths leads to problems such as water inrush, and gas/rock outbursts [99].

According to Clarke and Malone [11], sucker rod pumping systems operating in Eagle Ford also contain CO_2_ and H_2_S with concentrations ranging from 0 to 10% and 0 to 7%, respectively. This is an important contaminant to be considered since produced fluids and gases such as H_2_S in varying concentrations can have a significant impact on the material properties of polymer rod guides [53]. Moreover, as studied by Correa et al. [17] and Takacs [16], the presence of solid particles, such as sand from productive formations, causes abrasions which mainly occur on the guide’s surface. Therefore, the polymeric guide materials used in downhole operations should be corrosion and abrasion resistant and have good mechanical strength.

## 6. Recycling Methods for Polymer Rod Guides

Thermoplastic waste from sources such as the oil and gas industries is associated with having a large volume and a high-risk nature, causing societal, environmental, and economic concerns [100,101]. Polymeric waste from such diverse sources is also exposed to mechanical wear and weathering-induced changes that affect its chemical composition and physical characteristics [102]. Regulations have been put in place for the effective management of plastic waste through different methods such as recycling, reusing, or conversion to energy by processes such as pyrolysis [103]. Efforts have been made to increase post-use pathways, such as implementing plastic identification codes for effective recycling, which was introduced by the Society of the Plastics Industry (SPI) [101].

The material manufacturing costs of recycled sources could potentially be lower than those of virgin materials improving the economic aspect of the process [104] and recycled materials can sometimes be more optimal than virgin materials from a lifecycle assessment perspective [105]. However, recycling polymeric waste can be more expensive than other disposal alternatives, such as incineration and landfilling [106]. According to Wong et al. [106], plastic waste recycling can be divided into the following four categories:

1. Primary (commonly conducted in-plant) recycling or closed-loop recycling processes which reuse waste products from a single homogeneous source in their original form without any modification. It is advantageous due to lower energy consumption, reduced resource utilisation, low cost, and simple techniques [107,108].
2. Secondary recycling involves recovering plastic waste through mechanical processes. Mechanical recycling includes sorting, shredding, washing, and extruding post-consumer plastic [109]. Mwanza [108] and Grigore [110] indicated that mixing secondary recycled materials with virgin materials can yield excellent results, provided contaminants have been removed from the source.
3. Tertiary recycling or chemical recycling includes processes which produce monomers from waste polymer chains. Examples include gasification, pyrolysis, hydrocracking, depolymerisation, methanolysis, and aminolysis [111,112].
4. Quaternary (energy recovery) recycling is a process whereby plastic waste is incinerated and used for energy recovery to generate heat and electricity. It is generally employed when mechanical recycling is not feasible due to severe heterogeneity, which leads to separation difficulties, excessive contamination, or deteriorated polymeric properties.

Many complexities affect recycling methodologies, as reported by Thiounn et al. [101] including considerations for collection, sorting, and the need for pretreatment of polymers exposed to contaminants. Technical factors, such as chemical reactivity, also need to be taken into account [113]. Hence, for each recycling method technical, economic, and commercial viability should be assessed [114].

Primary recycling involves recycling of uncontaminated waste polymeric materials into the same “new” product through closed-loop processes. In secondary recycling, polymers undergo mechanical recycling processes in which the chemical structure of the polymer is retained during reprocessing [101]. The classification of polymeric waste is crucial for this process, as heterogeneous sources or different grades of plastic often phase-separate when mixed for recycling due to their high resistance to being reformed. Achieving an identical composition through the use of a homogeneous source is essential for effectively recycling plastic waste in mechanical recycling processes [115].

Pyrolysis is a type of chemical recycling in which the thermochemical decomposition of the organic and synthetic components of plastic occurs at high temperatures (500 °C to 800 °C) and in the absence of oxygen in order to produce fuel [116]. This process is, however, detrimental to public health and is often not regarded as a clean technology due to air emissions releasing toxic chemicals, and contaminated by-products (dioxins, mercury, heavy metals) leaching into groundwater and rivers [101,117]. The accumulation of plastic in landfills is a major problem and the use of incinerators can also cause severe environmental pollution [116]. Furthermore, oil converted from plastic waste is found to be less popular due to its low quality, waxing, bad odour, and deterioration in colour [118].

The last method, which does not result in any value-added product, involves incinerating polymers for energy recovery in the form of heat. This process can release toxic gases and residues, posing significant waste management and ecological concerns [114]. For instance, burning nylon for energy can emit hazardous smoke containing Hydrogen Cyanide (HCN), Carbon dioxide (CO_2_), Carbon monoxide (CO), and Ammonia (NH_3_) during combustion. It can result in the loss of ε-caprolactam in nylon rod guides, subsequently leading to a loss of economic value [119]. Hence, these recycling methods are not recommended for managing waste composite rod guides made of thermoplastics such as nylon. Evaluating the economic and environmental benefits, including the advantages and drawbacks of establishing a green supply chain can highlight the implications and strategic changes resulting from using recycled versus virgin materials [120].

Each recycling method has its own advantages depending on the type of polymeric waste, homogeneity, location, and level of contamination [114] as seen in Table 3. The best commercially beneficial method for used thermoplastic composite rod guides, which are a homogeneous source of waste and retain their polymeric chemical identity, is mechanical recycling. This method provides effective waste management as well as economic benefits. The steps involved include collection, sorting, washing, and grinding for efficient recycling [107,121]. By mixing mechanically recycled polymer with virgin material, the end product would yield comparable qualities to new materials [108,110]. In this case, the step of pre-treatment is crucial before undertaking the mechanical recycling process [101].

Based on the comparison between recycling methods for thermoplastic rod guides in Table 3, mechanical recycling, which became widespread in the 1970s [114], has the greatest potential to reduce waste generation while extending the lifecycle of polymeric materials [20]. This is the most suitable approach for managing used thermoplastic composite rod guides because primary recycling can only be applied to uncontaminated waste [12]. While tertiary and quaternary recycling methods, which can process contaminated waste, can require significant energy resources, cause loss of economic value due to polymer structure breakdown, and pose substantial environmental risks from toxic air emissions [23,30]. Additionally, these methods involve complex processes such as catalyst use, which negatively impacts commercialisation opportunities.

Mechanical recycling helps retain the polymer’s chemical structure and produces high quality products when adequate pre-treatment is performed. For example, studies by Al-Salem et al. [114] and Zia et al. [126] have demonstrated that mechanical recycling is the most economically and environmentally efficient method for thermoplastics, especially when blended with virgin material to maintain its properties. While challenges exist, such as contamination levels, degradation, and polymer chain alterations [114] including the loss of mechanical strength in recycled products, for homogenous wastes such as used composite rod guides, pre-treatment methods for contaminant removal can be adopted to produce high quality recycled products. Hence, mechanical recycling helps retain the economic value of composite rod guides while also reducing the environmental degradation caused by landfill accumulation and hazardous smoke from burning.

## 7. Identified Research Gaps

Despite the extensive industrial use of polymeric sucker rod guides, research on their performance and degradation under realistic downhole conditions remains limited. Several key knowledge gaps have been identified based on the current review:

- Lack of thermal degradation data: The existing literature mainly discusses mechanical wear and erosion, while systematic evaluation of thermal degradation at elevated downhole temperatures is absent. Understanding thermal stability and changes in mechanical properties with temperature is crucial for predicting the long-term performance of thermoplastic rod guides.
- Absence of microstructural and chemical characterisation: No published work has examined the microstructural or chemical changes in used rod guides through techniques such as SEM, FTIR, or DSC. These analyses are necessary to determine degradation pathways, fibre-matrix interfacial failure, and the extent of chemical attack caused by oilfield contaminants.
- Lack of a standardised testing framework: There is no established testing standard for evaluating polymeric rod guides under combined mechanical, thermal, and chemical stress. The absence of these benchmark testing parameters, such as wear rate, friction coefficient, or temperature limits, makes performance comparison between materials and field studies difficult.
- No defined recycling or reuse strategies: Used composite rod guides are currently discarded in landfills due to the absence of established recycling methods. There are no studies assessing their mechanical recyclability, potential reuse as feedstock, or environmental impact through a lifecycle perspective.
- Limited modelling and predictive approaches: No studies have attempted to model the wear behaviour, material degradation, and rod–tubing interaction using simulations or finite element methods. Therefore, development of such predictive tools would enable performance forecasting and design optimisation for various well conditions.

Addressing these gaps will provide essential data to improve the design, performance, and sustainability of polymeric rod guides, and will assist the oil and gas industry in advancing toward circular economy principles.

## 8. Challenges

### 8.1. Lack of Research and Development on Polymer Rod Guides

There are many studies involving research and development about increasing the efficiency and productiveness of oil and gas exploration using artificial lifting technologies [6,23,43]. However, amidst the available research, there are only a handful of studies indicating the significance of polymer rod guides as an important downhole component that enables the prevention of diverse system and equipment failures [10,22,24]. These polymers which are subjected to harsh environmental conditions are regularly replaced to ensure adequate performance in carrying out their downhole operations and functions [9].

The current practice adopted by oil and gas industries is to discard them in landfills because of the unavailability of research and development on the recyclability of used polymer rod guides. This is also a hazardous practice because polymeric material, such as nylon, releases harmful gases such as dioxins, nitrous oxide, and hydrogen cyanide [127] which can be disastrous during an event of bushfire. This is not only increasing environmental burden and economic losses but also contributing to polymeric waste generation which already has the lowest recovery rate at only 13% in Australia [128].

Therefore, polymeric wastes need to be reduced, reused, recycled, and recovered [129,130], including those produced from oil and gas exploration applications. While the cost, property degradation, and contamination associated with mechanical recycling affects plastic recovery rates [131], proper characterisation can enable introduction of technically viable recycling methods for polymeric rod guides. Moreover, while petroleum independent sustainable polymers from renewable resources are the most environmentally friendly options [132], it is also possible to introduce circular economy concepts by reusing existing high strength polymers which remains critical for this sector [133]. This also paves the way for the sustainable use of thermoplastic composites such as glass-fibre-reinforced polymers which have gained popularity due to their versatility and performance [134] and are known to undergo weathering ageing due to elevated temperature, exposure to moisture, and UV radiation causing degradation [135,136].

### 8.2. Lack of Characterisation Studies

There are a few important research studies dedicated to the acknowledgment of polymer rod guides as a critical tool in preventing some important failures such as rod string buckling or breakage [15,51], parted tubing [11,12,14], paraffin build-up [16], and corrosion failures [45]. Additionally, studies such as [8,10,24] majorly discuss the use of guides and the causes of its failures. However, in all these research studies, there is lack of a comprehensive failure analysis that will enable oil and gas industries to effectively recycle used polymeric rod guides. This is important because of the uncertainties and complexities involved in geological formations and conditions of mining environments [137].

The experiments described by Clemens at al. [8] and Permanschlager [15] demonstrate that weight and diameter reduction through the loss of polymeric material is the primary cause of rod guide failure. These studies ascertain that the only measure of rod guide failure is through calculation of EWV. Furthermore, studies such as [23] point out EWV while addressing functionality and applications of polymer guides. While one study [10] discusses the issue of rod guide disposals after a predefined time interval and another study [11] suggests the importance of material compatibility with well fluids and temperature rating, there are no research studies that contain an elaborate characterisation of used polymeric rod guides. This lack of in-depth chemical, morphological, thermal, and microstructural characterisation greatly inhibits the ability of oil and gas industries to recycle used polymer guides. Such studies are critical to understand the actual cause of failures, determine the extent of materials contamination, and evaluate its capability to be recycled without the risk of undergoing any gradual or catastrophic failures during downhole operations.

### 8.3. Wide Range of Polymeric Rod Guides Materials

The ongoing practice of simply shredding and disposing used composite polymer guides in landfills is causing significant environmental as well as economic drawbacks in the oil and gas industry [10,24]. Currently, around 300 metric tonnes of rod guide waste are generated every year in Australia and roughly 18,600 metric tonnes of rod guides are disposed annually around the world. While the challenge of limited studies on polymer guides and their causes of failure persists, another major challenge that needs to be addressed is the future of different thermoplastics that are used in manufacturing rod guides. A wide range of thermoplastic materials such as PPA, PA, PPS, and PEEK [16] reinforced with either glass or aramid fibres are used in fabricating rod guides to cater to different production applications, which include demanding well conditions such as high-water cuts, high temperatures, and presence of corrosive and abrasive particles. While studies have shown that reinforcement with glass fibres results in better property retention of thermoplastics [138], it is still critical to evaluate the effect of long-term exposure in an oil and gas well’s environment.

Glass-fibre-reinforced PA demonstrates improved stiffness and wear resistance under cyclic sliding conditions, although performance can be degraded by moisture ingress and hydrothermal ageing, which reduces toughness and increases frictional wear [20,56]. Glass-fibre-reinforced PPS provides excellent chemical resistance and dimensional stability in high-temperature and sour environments, making it a strong candidate where chemical attack and elevated temperatures are present [29]. On the other hand, glass-fibre-reinforced PEEK retains strength and creep resistance at elevated temperatures and under sustained loading, which supports its use in high-temperature wells [28]. Therefore, these studies show that glass fibre reinforcement substantially improves the mechanical and thermal resilience of polymers for rod guides, but that each polymer has distinct properties (moisture sensitivity, chemical attack, cost) that should support material selection for specific downhole conditions.

Therefore, due to the above mentioned challenges, recycling polymer from composite rod guides is very limited and rather scattered with no definitive outcome. This is despite the fact that recycling thermoplastics is practically and technologically easy [139], with mechanical recycling being the most developed with high industrial feasibility [140]. While there is a consensus among researchers that recyclability studies need to be carried out on all these polymeric materials so that the composite rod guides can be used and re-used for increased sustainability in the future, an ongoing significant drawback is that currently there are no failure or recyclability studies conducted on any of these popular polymer guide materials.

## 9. Research Opportunities

Commercial and Industrial Stream (C&I), which is accountable for 50% of the total polymeric waste in Australia, is inclusive of waste from the petroleum and coal product manufacturing sector (11.2%) which is liable for landfilling 77% of it [141]. This waste also comprises composite polymeric rod guides from oil and gas industries. While the low biodegradability of plastic waste affects its recycling potential in both large and small industries [142,143,144], there is still an unexplored scope for recycling plastic rod guides which has glass fibres. Moreover, it is to be noted that the cost of one rod guide is approximately AUD 10 to 50 depending on the type of material. For example, rubber guides are cheaper while composite thermoplastic guides with fibre reinforcements are expensive. Therefore, the relative cost of composite polymeric rod guides in comparison to sucker rods, which are priced between AUD 20 to 60, is very significant as each sucker rod is installed with an average of three to four polymer rod guides. With the expanding global plastic waste problems and annual recycling rates of only 9 to 15% in the market [145], many researchers have introduced solutions to address growing waste management issues and successfully studied various types of plastics, including thermoplastics from different sources. However, composite rod guides made of glass-fibre-reinforced thermoplastics, have not been researched in-depth and there are no studies on rod guide recycling technologies. While fibre-reinforced polymers are challenging to recycle and care must be taken to preserve the fibre length and orientation distribution [146]. If composite polymer rod guides are correctly recycled, it will contribute to global plastic waste management practices. Moreover, the mining industry globally is making an effort to improve mining productivity while conserving the environment [147]. Climate change and the escalating fossil fuel crisis point to the urgency required in transitioning to clean energy sources [148,149]. Therefore, there is a significant research and development scope for polymeric rod guides which is highlighted in the following sub-sections.

### 9.1. Degradation Mechanism of Polymeric Rod Guides

The failure of polymer guides has only been explored through the determination of its EWV. It was assumed by past researchers that rod guides fail to perform their functions once enough material from their EWV has worn out, making them inefficient in preventing contact between the tubing and the sucker rod and/or coupling. However, no research has ever been undertaken to carry out the characterisation of used polymer guides to examine their chemical, morphological, and thermal properties. It is known that polymer guides can suffer chemical degradation resulting from changes in their chemical structure after prolonged exposure to various contaminants and solvents. Furthermore, polymer guides can also have alterations to their morphological and thermal properties due to aggressive stroking movements and exposure to high downhole operating temperatures. Therefore, research can be performed on characterising used polymer guides which will give a broad understanding of failure mechanisms. Research outputs from failure and degradation mechanism studies will enable oil and gas industries to recycle the used polymer guides, which will have commercial benefits as well positive environmental impacts through the promotion of a circular economy.

### 9.2. Increased Recyclability of Used Polymer Rod Guides

Limited research and development activities have been performed to evaluate the recyclability of used polymer rod guides because oil and gas industries continue to dispose of them in landfills. This is critical as it is anticipated that oil and gas extraction and exploration will increase for the economic development of many countries. These polymeric materials can be recycled and used as feedstock in the manufacturing of new rod guides and this in turn can be utilised for the safe operation and use of around 55,000 oil and gas wells around the world. Hence, there is a wide scope for carrying out recycling studies on composite polymer guides through the determination of optimal mix ratios with virgin polymer. This will enable the industry to reuse the used guide materials as well as maintain their critical properties so that recycled guides can perform their intended functions for a longer usage time.

### 9.3. Study on Different Thermoplastics

As discussed in this review, there are a variety of thermoplastics that are used in manufacturing composite rod guides. Hence, there is a scope for researchers to study different thermoplastics used in rod guide manufacturing and carry out comparative analyses. This type of research will also assist the oil and gas industries in making researched decisions on the best material for their rod guide fabrication. Some of the important reasons for studying the different thermoplastics are (but not limited to) the following:

- To better understand their recyclability and compatibility with similar plastics.
- To help identify the potential for commingling with different plastics.
- To ensure the sustainability of sources for waste rod guide resources and support investments in the recycling industry.

Recycling waste plastics efficiently supports environmental conservation and resource preservation and incorporating them into petroleum-related processes offers a promising solution for transforming plastic waste into more valuable fuel products [150].

**Figure 12:**
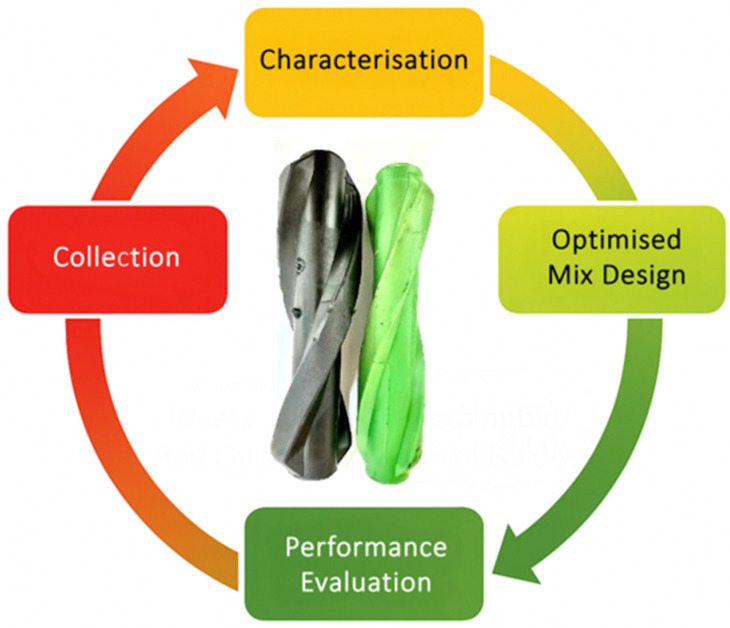
Circular economy implementation pathways for polymeric sucker rod guides with conceptual framework illustrating the collection, characterisation, optimised mix design, and performance evaluation stages of the recycling loop. Source: Author’s own conceptual design.

**Figure 13:**
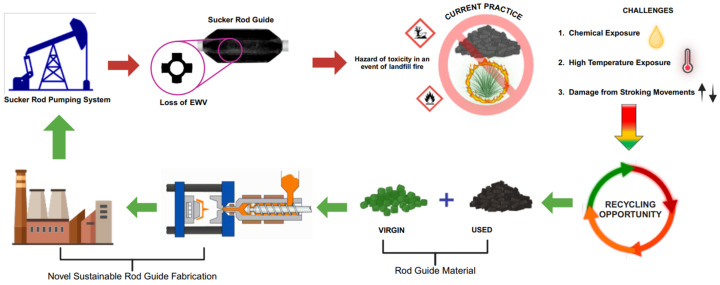
Material recovery and recycling process flow from current disposal practices toward re-fabrication using virgin and recycled polymer blends, demonstrating alignment with the EU Green Deal and UN SDGs 9 (Industry, Innovation, and Infrastructure) and 12 (Responsible Consumption and Production). Source: Author’s own conceptual design.

A circular economy framework was developed to illustrate potential material recovery and reuse pathways for polymeric sucker rod guides as depicted in Figure 12 and Figure 13 to contextualise the proposed sustainability approach. The conceptual loop in Figure 12 outlines the major steps involved including collection of used guides, material characterisation, optimised mix design for recycling, and performance evaluation of reprocessed components. Figure 13 expands this pathway into a process-level flow, highlighting the recycling opportunity for combining virgin and used polymeric materials in the fabrication of new rod guides. Through the combination of these frameworks, a closed-loop recovery system consistent with the principles of the EU Green Deal and UN Sustainable Development Goals (SDGs) 9 and 12, promoting sustainable production and responsible resource management within the oil and gas industry can be achieved.

Future research should focus on the lifecycle assessment (LCA) of recycled polymeric rod guides to quantify environmental and economic benefits over conventional disposal routes. Experimental validation of degradation mechanisms under controlled downhole conditions is also necessary to correlate laboratory findings with field performance. Furthermore, developing predictive models for Erodible Wear Volume (EWV) loss based on operating parameters, material composition, and environmental exposure would provide a scientific basis for optimising the design life and circular economy integration of sucker rod guides.

## 10. Concluding Remarks

This paper presents an extensive review of polymer rod guides, their uses, designs, materials, and failure mechanisms. This comprehensive review showed that polymeric guides are extensively used as a downhole tool to mitigate various types of system failures, and they reduce maintenance costs as well as production losses due to well downtime. This results in an annual 18,600 metric tonnes of polymeric waste generation from the disposal of used guides globally. Nevertheless, polymeric rod guides are one of the least researched and studied materials of artificial lift systems around the world. Prevailing research studies have very limited information on the cause of their failure and extremely limited research has been conducted to evaluate their recyclability. Due to such circumstances of the absence of data and information, hundreds of thousands of polymer guides continue to be disposed of in landfills.

The limited scientific evidence on their failure has greatly impacted the ability to recycle used rod guide materials. Furthermore, there are many different types of thermoplastics such as Polyphenylene Sulphide (PPS), Polyacrylamide (PAA), Polyamide (PA), and Polyether ether ketone (PEEK) that are used in manufacturing rod guides according to different well requirements and based on whether it is a Coal Seam Gas (CSG) or an oil well. These thermoplastics are also reinforced with glass fibres for enhanced material properties. However, there are some very critical challenges in recycling used polymer guides which have been identified. The unavailability of recyclability studies has posed economic as well as environmental drawbacks. This challenge arises from the lack of characterisation and failure studies. Moreover, with a wide range of thermoplastics being used to manufacture glass-filled composite rod guides, research output on the effect of downhole environments on different materials and their impact on reusability has not been investigated. Therefore, there are many unexplored research areas on polymeric rod guides as described in this review. Research and development in these critical areas would provide data and information necessary to help oil and gas industries around the world to recycle polymers used as rod guides and devise waste management strategies in line with their sustainability goals.

## Figures and Tables

**Table 1:** Different types of rod guides.

Sl. No	Type of Guide	Pictorial Illustration
1	Wheeled rod guides	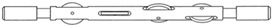
2	Snap-on guides	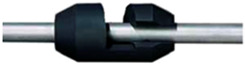
3	Moulded guides	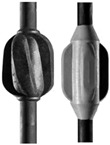

**Table 2:** Rod guide material property comparison with data from [16,26,63,66,67,68,69,70].

Sl. No	Material Property		Rod Guide Materials				
			PPA	PPS	PA		PEEK
					PA6	PA12	
1	Tensile Yield Strength		68.9–233 MPa	33.1–203 MPa	12.2–1500 MPa	12–140 MPa	180 MPa
2	Flexural Strength		103–326 MPa	53.1–339 MPa	29–800 MPa	120–185 MPa	265 MPa
3	Flexural Modulus		1.5–14.1 GPa	1.2–30.1 GPa	0.724–18.5 GPa	3.7–7.1 GPa	11.1 GPa
4	Izod Notched Impact Strength		0.37–3.74 J/cm	0.27–3.15 J/cm	0.370–3.36 J/cm	1.07–1.80 J/cm	0.214–11.4 J/cm
5	Hardness		114–125	114–123	80–121	109–110	85–109
6	Coefficient of Friction		0.220–0.290	0.150–0.350	0.150–0.610	0.02–0.67	0.15–0.4
7	Melting Temperature		246–340 °C	260–282 °C	178–368 °C	165–185 °C	342 °C
8	Glass Transition Temperature		95–135 °C	90 °C	5–67 °C	48.8 °C	143 °C
9	HDT at load at 1.8 MPa		155–320 °C	105–272 °C	65–290 °C	90–177 °C	≥280 °C
10	Density		1.34–1.75 g/cc	1.4–1.69 g/cc	1.20–1.67 g/cc	1.15–1.96 g/cc	1.50 g/cc
11	Moisture Absorption @ Saturation		4.2–4.5%	0.02%	1–8.2%	1–1.4%	0.18%
12	Resistance to	Hot Oiling	Yes	Yes	Yes	Yes	Yes
		Sweet Crude	Yes	Yes	Yes	Yes	Yes
		Sour Crude	Yes	Yes	No	No	Yes
		Sand	No	Yes	Yes	Yes	Yes
		Water	Yes	Yes	No	Yes	Yes
		Brine	No	Yes	No	Yes	Yes

**Table 3:** Comparison between different recycling methods.

Recycling Method	Steps Involved	Advantages	Disadvantages	Comment	Reference
Primary (In-Plant) Recycling	Collection of waste; Sorting; Direct reuse in manufacturing without altering the material	Low energy consumption; Minimal processing; Cost effective	Limited to clean, homogeneous waste	Suitable for uncontaminated in-plant waste; Most efficient in terms of energy and cost; Not suitable for used composite rod guides requiring pretreatment	[32,122]
Secondary (Mechanical) Recycling	Collection; Sorting; Cleaning; Shredding; Melting and extrusion into new products	Reduces the volume of waste; Moderate energy requirement; Extends lifecycle of the material	Degradation of polymer properties over multiple recycling cycles; Energy requirement for melting and reprocessing	Suitable for mixed or contaminated nylon waste; Is suitable for composite rod guides after cleaning/treatment procedures	[32,114]
Tertiary (Chemical) Recycling	Collection; Sorting; Depolymerization (hydrolysis, pyrolysis); Purification of monomers; Repolymerisation; Utilisation in new products	Recovers monomers for polymer production; Removes contaminants for heavily degraded mixed nylon waste	High energy consumption; Expensive; Complex processing	Suitable for heavily contaminated waste; Can cause air emission; Use of incinerators not environmentally friendly; Loss of economic value	[113,123]
Quaternary (Energy Recovery) Recycling	Collection; Sorting; Incineration for energy (heat/electricity)	Generates energy from waste; Reduces landfill	Not a recycling methodology; Releases toxic emissions; Loss of value of the material	Causes environmental concerns; Only used when contamination or heterogeneity prevents other recycling methods	[124,125]

## Data Availability

No new data were created or analyzed in this study. Data sharing is not applicable to this article.
